# Habitual Alcohol Intake and Risk of Atrial Fibrillation in Young Adults in Korea

**DOI:** 10.1001/jamanetworkopen.2022.29799

**Published:** 2022-09-02

**Authors:** Minju Han, So-Ryoung Lee, Eue-Keun Choi, JungMin Choi, Jaewook Chung, Sang-Hyeon Park, HuiJin Lee, Hyo-Jeong Ahn, Soonil Kwon, Seung-Woo Lee, Kyung-Do Han, Seil Oh, Gregory Y. H. Lip

**Affiliations:** 1 Department of Internal Medicine, Seoul National University Hospital, Seoul, Republic of Korea; 2 Department of Internal Medicine, Seoul National University College of Medicine, Seoul, Republic of Korea; 3 Department of Medical Statistics, College of Medicine, Catholic University of Korea, Seoul, Republic of Korea; 4 Statistics and Actuarial Science, Soongsil University, Seoul, Republic of Korea; 5 Liverpool Centre for Cardiovascular Science, University of Liverpool and Liverpool Heart and Chest Hospital, Liverpool, United Kingdom; 6 Department of Clinical Medicine, Aalborg University, Aalborg, Denmark

## Abstract

**Importance:**

Guidelines recommend that all risk factors for early-onset atrial fibrillation, including lifestyle factors, be proactively managed, considering the poor prognosis of the disease. Not much is known about the association of cumulative alcohol intake with the risk of atrial fibrillation in young adults aged 20 to 39 years, especially among heavy drinkers.

**Objective:**

To explore the association of alcohol consumption with the risk of incident atrial fibrillation in young adults.

**Design, Setting, and Participants:**

Using the National Health Insurance Service database, a nationwide population-based cohort study of adults aged 20 to 39 years without prior atrial fibrillation who underwent 4 serial annual health examinations between 2009 and 2012 was conducted. The cumulative alcohol consumption burden over 4 years was calculated by assigning 1 point to more than moderate drinking (≥105 g of alcohol per week) each year. Additionally, a semiquantitative cumulative burden was calculated by assigning 0, 1, 2, and 3 points to non, mild (<105 g per week), moderate (105-210 g per week), and heavy (≥210 g per week) drinking, respectively. Data were analyzed from May to June 2021.

**Exposure:**

Amount of alcohol intake in 4 years.

**Main Outcomes and Measures:**

The primary outcome was incident atrial fibrillation during the follow-up period.

**Results:**

A total of 1 537 836 participants (mean [SD] age 29.5 [4.1] years, 1 100 099 [71.5%] male) were included in the final analysis. According to the 4-year cumulative burden of alcohol consumption stratified by moderate to heavy drinking, 889 382 participants (57.8%) were in the burden 0 group, 203 374 participants (13.2%) in the burden 1 group, 148 087 participants (9.6%) in the burden 2 group, 144 023 participants (9.4%) in the burden 3 group, and 152 970 participants (9.9%) in the burden 4 group. During a median (IQR) follow-up of 6.13 (4.59-6.48) years, atrial fibrillation was newly diagnosed in 3066 participants (0.36 per 1000 person-years). Participants with a cumulative burden of 4 points who continued more than moderate drinking for 4 years showed a 25% higher risk of atrial fibrillation compared with 0-point participants who kept non-to-mild drinking over 4 years (adjusted HR, 1.25; 95% CI, 1.12-1.40). In a semiquantitative analysis, participants who sustained heavy drinking for 4 consecutive years were associated with a 47% higher atrial fibrillation risk than those who remained nondrinkers over 4 years (aHR, 1.47, CI 1.18-1.83).

**Conclusions and Relevance:**

Persistent moderate to heavy drinking and higher cumulative alcohol consumption burden might increase the risk of atrial fibrillation even in young adults aged 20 to 39 years.

## Key Points

**Question:**

Is high alcohol intake associated with the risk of atrial fibrillation in young adults?

**Findings:**

In this cohort study including 1 537 836 adults aged 20 to 39 years, an up to 25% higher risk of incident atrial fibrillation was associated with a higher cumulative alcohol burden for 4 years, and up to 47% with sustained heavy drinking over 4 years.

**Meaning:**

Persistent moderate to heavy drinking and higher cumulative alcohol consumption burden were associated with an increased risk of atrial fibrillation in young adults aged 20 to 39 years, suggesting the need for education on the risk of atrial fibrillation in young heavy drinkers.

## Teaser

This cohort study evaluates the risk of atrial fibrillation associated with habitual alcohol intake in young adults in Korea.

## Introduction

Despite its lower prevalence in younger people, atrial fibrillation (AF) and AF-related complications are still associated with poor clinical outcomes.^1,2,3,4^ The recurrence rate of AF reaches 50% despite treatments including cardioversion and antiarrhythmic drugs.^5^ Considering the long life expectancy of younger people, awareness of AF prevention is necessary.

Alcohol is a well-known risk factor for AF development.^6,7,8^ Several studies have been conducted on the mechanism by which alcohol induces AF. One of the potential mechanisms is tachycardia from alcohol-induced autonomic imbalance leading to AF.^9^ Changes in cardiac structure and function including cardiomyopathy and atrial remodeling are also thought to have an effect.^10,11,12,13^ It has also been suggested that hypertension, obesity,^14^ and heart failure^10,15^ linked to excessive alcohol consumption are risk factors for incident AF.

Although heavy drinking among young adults, especially those between 18 to 29 years, is a serious social issue that many countries are facing,^16,17,18^ few studies on the association of AF and alcohol have been conducted in this population. Even among studies that have explored AF in this population, the age criteria for younger age were under 65 years,^19^ 60 years,^20^ and 45 years.^21^ Furthermore, the proportion of participants between 18 and 29 years was small.

Heavy drinking is a modifiable factor associated with risk for AF, since it can be improved with education and awareness. Using a nationwide population-based cohort, we investigated the outcomes of a 4-year cumulative burden of alcohol consumption on the risk of incident AF in young adults aged 20 to 39 years.

## Methods

This cohort study was exempted from review and the need for informed consent by the Seoul National University Hospital Institutional Review Board because anonymized and deidentified information was used for this analysis. The analysis was conducted from May to June 2021. This study followed Strengthening the Reporting of Observational Studies in Epidemiology (STROBE) reporting guideline.

### Data Source

We used the Korean National Health Information Database (NHID), which holds all data from inpatient and outpatient medical claims, including prescriptions, procedures and surgery records, and information on insurance premium payment.^22^ The Korean National Health Insurance System (NHIS) is compulsory for Korean citizens, with more than 51 million subscribers as of 2019.^23^ A national health examination is conducted annually or biennially for citizens older than 20 years. In addition to physical measurements and laboratory tests, a self-reported questionnaire is obtained.

### Study Population

**Figure 1.**
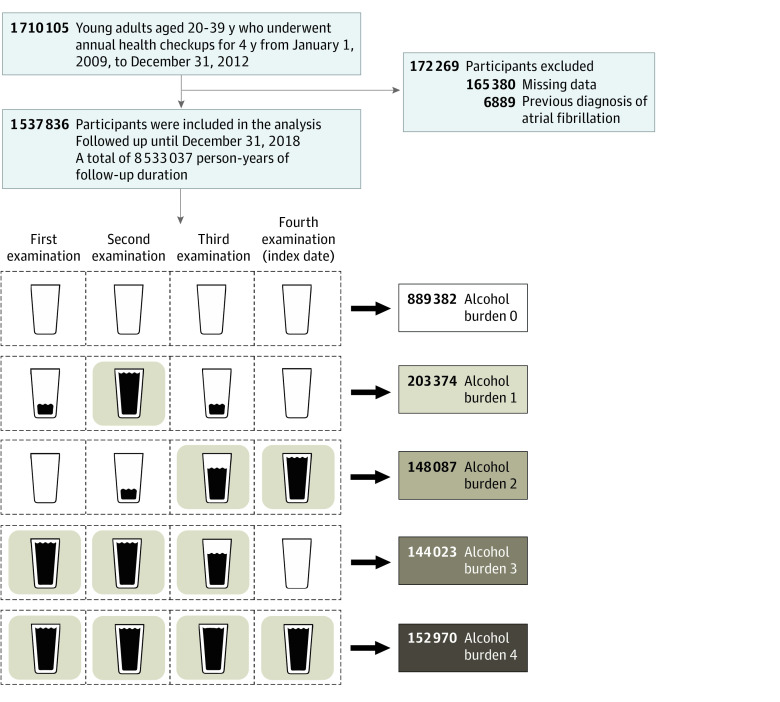
Flowchart of the Study

A flowchart of the enrollment is presented in Figure 1. Young adults aged between 20 and 39 years who underwent 4 consecutive NHIS health examinations from 2009 to 2012 were included. Participants with prevalent AF before the last (fourth) health examination and those with missing values were excluded.

### Definition of Cumulative Burden of Alcohol Consumption

The self-reported questionnaire and the calculation regarding the alcohol content in standard drinks are presented in the eMethods in the Supplement. Briefly, the calculated alcohol content in 1 standard drink was 7.5 g.^7^ We defined less than 105 g (7.5 g × 14 drinks), more than 210 g, and 105 g to 210 g of weekly alcohol consumption as mild, heavy, and moderate drinking, respectively.

To operationally define the cumulative burden of alcohol, we assigned 1 point to each year of moderate to heavy drinking (>105 g per week). Therefore, participants were categorized into 5 groups according to their alcohol burden over 4 years (0, 1, 2, 3, and 4).

To evaluate the dose-response association between the amount of alcohol consumption and risk of AF, a more stratified scoring for alcohol burden was conducted. The novel semiquantitative cumulative alcohol consumption burden was calculated by assigning each 1, 2, and 3 points for mild, moderate, and heavy drinking (mild drinking, <105 g per week, moderate, 105-210 g per week, and heavy, ≥210 g per week). The participants were then categorized into 13 groups according to 4 years’ total points (0-12). For the detailed study design, refer to eFigure 1 in the Supplement.

### Covariates

Participants’ age, sex, underlying comorbidities, body mass index (BMI), smoking status, physical activity, and income level were included as covariates. All covariates were identified according to the information collected during the index (fourth) health examination.

Comorbidities were identified using the operational definitions according to the *International Statistical Classification of Diseases and Related Health Problems, Tenth Revision (ICD-10) *codes. Detailed definitions are provided in eTable 1 in the Supplement. We defined obesity as BMI greater than 25; BMI is calculated as weight in kilograms divided by height in meters squared.^24^ Smoking status and physical activity were assessed using a self-reported questionnaire, while regular physical activity was defined as moderate intensity exercise more than 5 times or vigorous intensity more than 3 times a week.^25^ Participants who paid the bottom 20% of health insurance premiums were defined as the low-income group.

### Study Outcome and Follow-up

The primary end point of the study was the diagnosis of new-onset AF during the follow-up. We ascertained AF using *ICD-10* codes I480 to I484 and I489 that were claimed at least once during hospitalization or twice in outpatient visits.^22^ Recognizing AF by *ICD-10* codes was validated in a study by Lee et al,^26^ where the positive predictive value of defining AF by *ICD-10* codes reached 94.1%. Participants were followed up from the index date (the last examination) to the date of new-onset AF, death, or the end of the follow-up (December 31, 2018), whichever came first.

### Statistical Analysis

Continuous variables are expressed as the mean and SD, while categorical variables are presented as numbers (percentages). A 1-way analysis of variance and χ^2^ tests were conducted to evaluate the baseline differences among groups.

The crude incidence rate (IR) of new-onset AF was calculated as the number of events per 1000 person-years (PYs). The cumulative incidence of AF was analyzed using survival analysis and log-rank test.

Using a multivariable Cox regression model, we analyzed the association between cumulative alcohol burden and the risk of AF. A model adjustment was made for covariates of age, sex, comorbidities, including hypertension, diabetes, dyslipidemia, heart failure, chronic obstructive pulmonary disease, thyroid disease, peripheral artery disease, prior myocardial infarction, prior ischemic stroke, sleep apnea, chronic kidney disease, BMI, smoking status, regular physical activity, and low income. The risks for AF are presented as hazard ratios (HRs) and 95% CIs for different cumulative alcohol burden groups. The level of significance was set at .05, and all analyses were 2-sided. We used SAS version 9.4 (SAS Institute) for statistical analyses. Analyses were conducted from May to June 2021.

#### Subgroup Analyses

We performed subgroup analyses and interaction tests to evaluate the potential outcomes of age, sex, comorbidities, BMI, smoking, physical activity, and low income. For subgroup analyses, we used multivariable Cox proportional hazards regression models. The analyses were 2-sided, and a *P* value of less than .10 was considered significant.

#### Sensitivity Analysis

To provide complementary analysis for healthy young adults, we conducted a sensitivity analysis for those without prior heart failure, myocardial infarction, and ischemic stroke. Participants were censored when these cardiovascular adverse events occurred. Apart from our newly defined categorization, we have performed additional analysis classifying participants according to the existing US drinking scale.

## Results

A total of 1 537 836 participants (mean [SD] age 29.5 [4.1] years, 1 100 099 [71.5%] men) were included in the final analysis (Figure 1). According to the 4-year cumulative burden of moderate to heavy drinking, 889 382 participants (57.8%) were in the burden 0 group, 203 374 participants (13.2%) in the burden 1 group, 148 087 participants (9.6%) in the burden 2 group, 144 023 participants (9.4%) in the burden 3 group, and 152 970 participants (9.9%) in the burden 4 group. Baseline characteristics of the study population are presented in Table 1. The proportions of men and current smokers were significantly higher in the groups with higher cumulative alcohol burden. The prevalence of hypertension, dyslipidemia, and obesity tended to be higher in higher alcohol burden groups.

**Table 1.** Baseline Characteristics of the Study Population

Characteristic	No. (%)						*P* value
	Total (N = 1 537 836)	4-y Cumulative burden of moderate to heavy drinking					
		0 (n = 889 382)	1 (n = 203 374)	2 (n = 148 087)	3 (n = 144 023)	4 (n = 152 970)	
Age, y							
Mean (SD)	29.54 (4.07)	29.44 (4.15)	29.26 (4.09)	29.51 (3.99)	29.85 (3.89)	30.23 (3.75)	<.001
20-29	758 589 (49.33)	443 576 (49.87)	106 440 (52.34)	74 367 (50.22)	67 863 (47.12)	66 343 (43.37)	<.001
30-39	779 247 (50.67)	445 806 (50.13)	96 934 (47.66)	73 720 (49.78)	76 160 (52.88)	86 627 (56.63)	
Sex							
Male	1 100 099 (71.54)	529 317 (59.52)	164 050 (80.66)	129 024 (87.13)	131 883 (91.57)	145 825 (95.33)	<.001
Female	437 737 (28.46)	360 065 (40.48)	39 324 (19.34)	19 063 (12.87)	12 140 (8.43)	7145 (4.67)	
Smoking							
Never	737 186 (47.94)	562 281 (63.22)	80 389 (39.53)	42 648 (28.80)	30 600 (21.25)	21 268 (13.90)	<.001
Former	226 450 (14.73)	103 265 (11.61)	36 130 (17.77)	28 770 (19.43)	28 316 (19.66)	29 969 (19.59)	
Current	574 200 (37.34)	223 836 (25.17)	86 855 (42.71)	76 669 (51.77)	85 107 (59.09)	101 733 (66.51)	
Underlying comorbidities							
Hypertension	117 935 (7.67)	47 930 (5.39)	16 264 (8)	14 541 (9.82)	17 323 (12.03)	21 877 (14.3)	<.001
Dyslipidemia	139 895 (9.1)	69 112 (7.77)	19 212 (9.45)	15 723 (10.62)	16 650 (11.56)	19 198 (12.55)	<.001
Chronic obstructive pulmonary disease	42 780 (2.78)	26 436 (2.97)	5338 (2.62)	3823 (2.58)	3575 (2.48)	3608 (2.36)	<.001
Sleep apnea	2021 (0.13)	921 (0.1)	332 (0.16)	258 (0.17)	242 (0.17)	268 (0.18)	<.001
Thyroid disease	25 048 (1.63)	18 344 (2.06)	2584 (1.27)	1576 (1.06)	1334 (0.93)	1210 (0.79)	<.001
Diabetes	33 279 (2.16)	15 906 (1.79)	4500 (2.21)	3990 (2.69)	4125 (2.86)	4758 (3.11)	<.001
Prior myocardial infarction	946 (0.06)	498 (0.06)	149 (0.07)	133 (0.09)	95 (0.07)	71 (0.05)	<.001
Heart failure	1047 (0.07)	592 (0.07)	159 (0.08)	90 (0.06)	101 (0.07)	105 (0.07)	.33
Peripheral arterial disease	11 093 (0.72)	6633 (0.75)	1456 (0.72)	999 (0.67)	980 (0.68)	1025 (0.67)	<.001
Chronic kidney disease	5404 (0.35)	3738 (0.42)	624 (0.31)	390 (0.26)	315 (0.22)	337 (0.22)	<.001
Prior stroke	929 (0.06)	540 (0.06)	129 (0.06)	107 (0.07)	85 (0.06)	68 (0.04)	.036
Regular exercise	272 060 (17.69)	150 289 (16.9)	38 075 (18.72)	28 428 (19.2)	27 216 (18.9)	28 052 (18.34)	<.001
Body mass index^a^							
Mean (SD)	23.62 (4.04)	23.05 (3.66)	23.98 (3.59)	24.33 (3.56)	24.61 (3.52)	24.85 (6.34)	<.001
Obesity (BMI ≥25)	499 408 (32.47)	237 406 (26.69)	72 334 (35.57)	58 434 (39.46)	61 442 (42.66)	69 792 (45.62)	<.001
Low income	49 467 (3.22)	36 257 (4.08)	4953 (2.44)	3116 (2.1)	2637 (1.83)	2504 (1.64)	<.001
Glomerular filtration rate, mean (SD), mL/min/1.73m^2^	102.11 (70.67)	102.06 (65.4)	102.33 (76.49)	101.97 (75.24)	102.38 (79.77)	101.95 (78.06)	.22
Blood pressure, mean (SD), mm Hg							
Systolic	119.08 (12.8)	116.75 (12.43)	120.18 (12.48)	121.75 (12.42)	123.23 (12.51)	124.63 (12.55)	<.001
Diastolic	74.93 (9.22)	73.39 (8.92)	75.56 (9)	76.66 (9.06)	77.68 (9.15)	78.75 (9.26)	<.001
Glucose, mean (SD), mg/dL	92.16 (16.33)	90.91 (15.32)	92.58 (16.48)	93.63 (17.8)	94.4 (17.61)	95.39 (18.16)	<.001
Total cholesterol, mean (SD), mg/dL	189.62 (34.91)	186.91 (34.2)	190.65 (34.77)	192.71 (36.04)	194.46 (35.82)	196.42 (35.51)	<.001
High-density lipoprotein, mean (SD), mg/dL	56.07 (19.12)	56.34 (18.52)	55.51 (18.54)	55.38 (17.41)	55.7 (17.9)	56.25 (25.01)	<.001
Low-density lipoprotein, mean (SD), mg/dL	109.36 (40.59)	109.04 (37.22)	110.17 (44.87)	110.22 (42.54)	109.77 (45.13)	108.92 (46.49)	<.001
Follow-up duration, mean (SD), y	5.55 (1.17)	5.53 (1.18)	5.53 (1.18)	5.57 (1.16)	5.6 (1.15)	5.6 (1.15)	<.001

SI conversion factors: To convert glucose to millimoles per liter, multiply by 0.0555. To convert high-density lipoprotein, low-density lipoprotein, and total cholesterol to millimoles per liter, multiply by 0.0259.

^a^ Body mass index is calculated as weight in kilograms divided by height in meters squared.

### Four-Year Cumulative Alcohol Consumption Burden and the Risk of Incident AF

**Figure 2.**
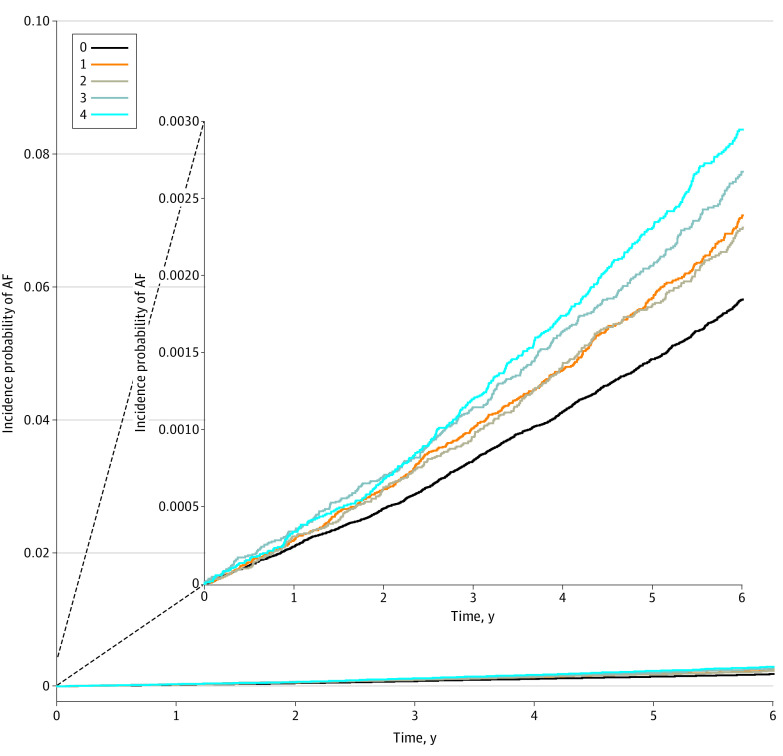
Cumulative Incidence Curves of Atrial Fibrillation (AF) According to 4-Year Alcohol Burden Participants with a higher cumulative burden of alcohol consumption during the 4-year period showed a higher incidence rate for atrial fibrillation than those who sustained non-to-mild drinking (burden 0).

**Figure 3.**
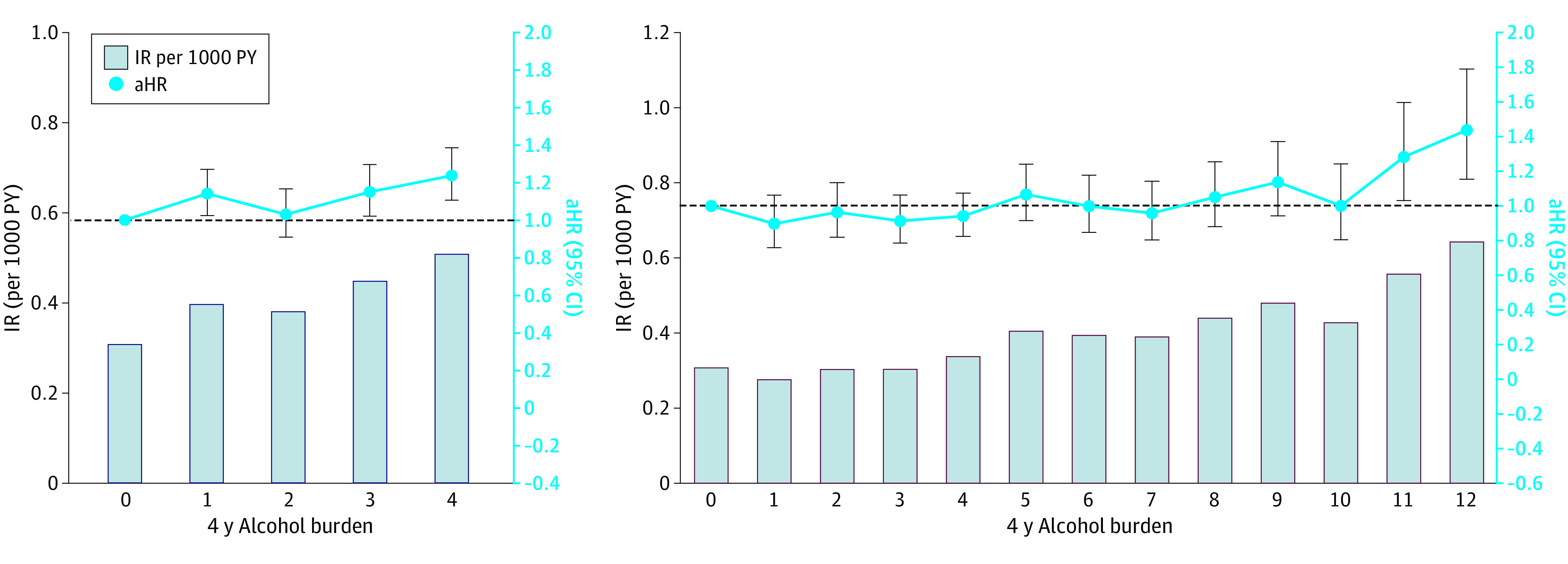
The Risk of Atrial Fibrillation According to 4-Year Alcohol Burden and 4-Year Cumulative Amount of Alcohol Consumption On the left, when participants are divided into 5 groups according to their 4-year alcohol burden (alcohol burden defined as >105 g/wk), higher burden groups show a higher incidence rate for AF than burden 0 group. On the right, when participants are divided into 13 groups according to their 4-year cumulative amount of alcohol consumption, calculated by assigning 1, 2, and 3 points for mild, moderate, and heavy drinking, respectively (mild drinking, <105 g per week; moderate, 105-210 g per week; and heavy, ≥210 g per week), groups with more than 10 points associate with higher incidence rate for AF. aHR indicates adjusted hazard ratio; IR, incidence rate; PY, person-year.

During a mean (SD) of 5.6 (1.2) years of follow-up duration (8 533 037 PYs), 3066 received a diagnosis of new-onset AF (IR, 0.36 per 1000 PY). The number of events, crude IR, unadjusted HRs, and curves for the incidence probability of AF according to cumulative alcohol burden are presented in Table 2 and Figure 2. Generally, participants with a higher cumulative burden of alcohol during the 4-year period showed a higher IR for AF than those with no burden. Adjusted HRs are presented in Table 2 and Figure 3A. Participants with burdens 1, 3, and 4 were associated with a 14%, 16%, and 25% increased risk of AF compared with the 0 group, respectively (Table 2 and Figure 3A). A cubic spline curve showing the association between the total amount of alcohol consumed per week at the 4 time points when the questionnaires were filled out and the incidence of AF is presented in eFigure 2 in the Supplement. Alcohol consumption between 700 g and 4200 g, which is approximately 90 to 560 standard drinks according to our categorization, and 50 to 300 standard drinks according to US standards, was associated with an increased risk of atrial fibrillation. The hazard ratio was not significant when the alcohol consumption was greater than 4200 g, presumably because the number of corresponding participants was too small.

**Table 2.** The Risk of AF According to the 4-Year Alcohol Burden and 4-Year Cumulative Amount of Alcohol Consumption

Alcohol consumption	Participants, No.	Participants with AF, No.	IR per 1000 person-years	HR (95% CI)	
				Crude	Adjusted model^a^
Alcohol intake ≥105 g/wk (moderate to heavy drinking) at the index date					
No	1 157 234	2104	0.33	1 [Reference]	1 [Reference]
Yes	380 602	962	0.45	1.38 (1.28-1.49)	1.13 (1.05-1.23)
* P* value	NA	NA	NA	<.001	.002
The 4-y alcohol burden (1 point to each moderate to heavy drinking [>105 g/wk] during 4 consecutive years)					
0	889 382	1512	0.31	1 [Reference]	1 [Reference]
1	203 374	445	0.4	1.29 (1.16-1.43)	1.14 (1.03-1.27)
2	148 087	313	0.38	1.24 (1.09-1.40)	1.04 (0.91-1.17)
3	144 023	361	0.45	1.45 (1.30-1.63)	1.16 (1.03-1.31)
4	152 970	435	0.51	1.65 (1.48-1.83)	1.25 (1.12-1.40)
* P* value	NA	NA	NA	<.001	<.001
Amount of alcohol consumption at the index date					
0	485 221	838	0.31	1 [Reference]	1 [Reference]
1 (<105 g/wk)	672 013	1266	0.34	1.10 (1.00-1.20)	0.97 (0.88-1.06)
2 (105-210 g/wk)	237 069	557	0.42	1.35 (1.21-1.50)	1.04 (0.93-1.16)
3 (≥210 g/wk)	143 533	405	0.51	1.63 (1.45-1.84)	1.22 (1.08-1.38)
* P* value	NA	NA	NA	<.001	<.001
The 4-y cumulative amount of alcohol consumption					
0	246 284	424	0.31	1 [Reference]	1 [Reference]
1	132 566	204	0.28	0.90 (0.76-1.06)	0.90 (0.76-1.07)
2	133 208	225	0.31	0.99 (0.84-1.16)	0.97 (0.82-1.14)
3	173 593	294	0.31	0.99 (0.85-1.15)	0.92 (0.80-1.07)
4	272 954	509	0.34	1.10 (0.97-1.25)	0.96 (0.84-1.09)
5	135 620	305	0.41	1.32 (1.14-1.53)	1.08 (0.93-1.26)
6	107 017	235	0.40	1.28 (1.09-1.50)	1.02 (0.86-1.20)
7	89 303	195	0.39	1.27 (1.07-1.50)	0.98 (0.82-1.16)
8	77 356	191	0.44	1.42 (1.20-1.69)	1.07 (0.90-1.28)
9	60 686	163	0.48	1.55 (1.30-1.86)	1.16 (0.96-1.40)
10	45 606	109	0.43	1.39 (1.12-1.71)	1.02 (0.83-1.27)
11	34 096	106	0.56	1.80 (1.46-2.23)	1.31 (1.05-1.63)
12	29 547	106	0.64	2.08 (1.68-2.57)	1.47 (1.18-1.83)
* P* value	NA	NA	NA	<.001	.002

Abbreviations: AF, atrial fibrillation; IR, incidence ratio; HR, hazard ratio; NA, not applicable.

^a^ The adjusted model was adjusted for age, sex, diabetes, hypertension, dyslipidemia, chronic obstructive pulmonary disease, sleep apnea, thyroid disease, myocardial infarction, heart failure, peripheral artery disease, chronic kidney disease, prior stroke, body mass index, smoking, performing regular exercise, and low income.

### Semiquantitative Cumulative Alcohol Consumption Burdens and the Risk of Incident AF

Calculating the semiquantitative cumulative alcohol consumption burden, participants who sustained heavy drinking over 4 years (burden 12) had a higher risk of incident AF by 47% than those who sustained nondrinking (burden 0) (Table 2 and Figure 3B). Participants with burden 11, who mostly continued heavy drinking but reported moderate drinking for a year, were associated with a 31% higher AF risk than burden 0 group (Table 2 and Figure 3B).

### Subgroup Analyses

The results of subgroup analyses according to participants’ age, sex, comorbidities including hypertension, diabetes, dyslipidemia, heart failure, chronic obstructive pulmonary disease, thyroid disease, peripheral arterial disease, myocardial infarction, ischemic stroke, sleep apnea, chronic kidney disease, BMI less than 25, BMI 25 or greater, smoking, regular exercise, and low income are presented in eTable 2 in the Supplement. There were no significant interactions between various subgroups and the associations between cumulative alcohol consumption burden and the risk of AF, except for the sex subgroup (*P *for interaction = .07). The main results were consistently observed only in men.

### Sensitivity Analysis

We analyzed the association between cumulative alcohol consumption burden and incident AF in apparently healthy young adults without a history and new occurrence of heart failure, myocardial infarction, or ischemic stroke during follow-up. The results were consistent with the main results. The risk of AF was higher in the burden 1 (HR, 1.15; 95% CI, 1.03-1.29), 3 (HR, 1.18; 95% CI, 1.04-1.34), and 4 groups (HR, 1.22, HR, 1.08-1.38) than in the burden 0 group (eTable 3 in the Supplement). When classification of participants’ drinking habit and cumulative alcohol consumption was done according to the existing US drinking scale, heavy drinking in the index year and more than moderate drinking for 3 or more years out of the screened 4 years was associated with increased risk of atrial fibrillation in men. However, the association was not clear in women (eTable 4 in the Supplement).

## Discussion

This cohort study investigated the association of cumulative alcohol consumption burden on the risk of incident AF in young adults. The principal findings of this study were: (1) the risk of AF was higher by 25% in participants who maintained moderate to heavy drinking for 4 years compared with those who sustained no-to-mild drinking; (2) persistent heavy drinking across 4 years was associated with a higher risk of AF by 47% compared with persistent nondrinking; and (3) a positive correlation between high cumulative alcohol consumption and higher risk of AF was consistently observed in apparently healthy young adults. These findings do not come as a surprise, since no clear interaction between alcohol and age has been reported from previous studies.

The prevalence and incidence of AF worldwide are increasing, as are the related health care costs.^27,28,29^ The most likely reason for the increasing prevalence of AF is the growing aging population, since age is a critical risk factor for AF and AF-related complications.^1,3,30,31,32^ AF is also associated with various cardiovascular risk factors and comorbidities, and multimorbidity is common among patients with AF, contributing to complications such as stroke and heart failure.^33,34,35^

Drinking is one of the risk factors of AF even among young people.^6,7,8^ Several mechanisms by which alcohol triggers AF include stimulation of the sympathetic nervous system that promotes adrenaline secretion, parasympathetic modulation of autonomic tone, and slowing of interatrial electrical conduction concomitant with a shorter atrial refractory period resulting in reentry.^13^ Structural changes in atria such as left atrial enlargement^11^ and atrial tissue fibrosis^13^ have also been proposed as possible pathophysiology. Although some studies^6,7,8,36,37^ have already revealed the association of excessive drinking with increased risk of AF, a few studies^38,39^ have recently paid attention to the threshold amount of drinking or drinking habits associated with the risk of AF. On the other hand, although some studies^40,41^ reported cardiovascular-protective association of mild or moderate drinking, each of these studies used different criteria to define the amount of drinking. Therefore, there has been no consensus on how much alcohol should be considered low- or high-risk drinking.^42^ We further consolidated these associations between alcohol intake and AF risk. The strength of this study lies in the study design, in which we enrolled a large number of young adults who had undergone 4 yearly health checkups. In light of the vast amount of data, we could evaluate the participants’ cumulative burden of moderate to heavy drinking over 4 years. Compared with previous studies with only cross-sectional estimation of the participants’ alcohol consumption status, our study could evaluate the participants longitudinally.

Another major novelty of our study is the application of 2 different concepts of cumulative alcohol burden. Analyzing the association between alcohol burdens that were defined by the 2 different methods and risk of AF, we confirmed that both persistent moderate to heavy drinking for 4 years and semiquantitative cumulative alcohol consumption burden higher than 10 points was significantly associated with a higher risk of AF. Participants in these groups (11 and 12 points) consumed over 2.5 points of alcohol per year on average, where 2.5 points of alcohol consumption indicated more than moderate (105-210 g) but less than heavy drinking (≥210 g). Alcohol intake in this range might represent a new threshold for average alcohol content over a 4-year period that increases AF risk and could be used as a reference in subsequent longitudinal studies.

Alcohol consumption among younger people is a global problem that has drawn increasing attention. According to a World Health Organization report,^17^ 13.5% of the total deaths among those 20 to 39 years old are related to alcohol consumption. Young adults (20-24 years) account for 48.5% of heavy episodic drinkers among all drinkers.^18^ To the best of our knowledge, the present study included the largest number of young adults as participants, especially those between 20 and 39 years of age. Given that AF induces fatal complications, including stroke,^43^ the prognosis is worse when AF is diagnosed at an early age.^3^ As the risk of AF and stroke is lowered by alcohol abstinence under various circumstances,^44,45^ young adults should be educated about the risk of AF and its association with drinking. Lee et al^44^ reported that alcohol abstinence in patients with AF reduced the risk of ischemic stroke, and a study of alcohol abstinence by Voskoboinik et al^45^ demonstrated a reduction in the recurrence and total disease burden in patients with AF. Choi et al^46^ found that the risk of AF was reduced with alcohol abstinence in patients who received a new diagnosis of diabetes. Addressing excessive alcohol consumption is part of an integrated care approach to AF care,^47^ and is associated with improved clinical outcomes.^48,49^

### Limitations

This study has limitations. First, because diagnostic codes of NHID are claimed by health care practitioners for medical billing and reimbursement and not for research, a disparity between the diagnostic code and actual diagnosis may exist because of contamination or coding inaccuracy. There is also the possibility of overestimation or underestimation of diagnoses, since the analyses are based on novel operational definitions.^8,22^ Second, this study was conducted only on an Asian population, so caution is required when applying the study results to other ethnicities. Third, alcohol consumption itself could be a factor associated with risk for other AF risk factors. The baseline characteristics of the participants in the moderate to heavy drinking burden group confirmed that there were more comorbidities such as hypertension, diabetes, and dyslipidemia among these people. Participants’ BMIs were also higher in the higher-burden groups. Although we went through adjustments to correct the influence of different comorbidities and body measurements, the severity of comorbidity or comorbidities’ management level might not have been reflected in the correction. Fourth, alcohol intake was surveyed using a self-reported questionnaire, in which a recall bias may have occurred. Nevertheless, this method has already been adopted by numerous studies in which meaningful research results were obtained.^7,37,39,44^ Fifth, the proportion of drinkers, particularly of moderate to heavy drinkers, was fundamentally too small in women. Therefore, it is not possible to conclusively state the association between alcohol consumption and AF in young women according to the results of this cohort alone. Sixth, the participants for annual health examination in Korea are employer-provided policyholders (workers and employers of all workplaces and public officials and school employees). Additionally, those who did not miss out on health checkups 4 times in a row are more likely to be interested in their health. Therefore, it is a limitation of our study that there may be a selection bias in the study population and might not represent the entire young population. Additionally, our analysis did not consider a change in drinking habits. Therefore, it cannot be concluded from the results of our study alone that drinking cessation reduces the risk of AF. People who drink heavily for 4 consecutive years are probably more likely to drink heavily during rest of their lives, and prolonged exposure to large amounts of alcohol during young ages might lead to left atrial remodeling, a well-known mechanism by which alcohol triggers atrial fibrillation. Further research is required to answer these issues that were not explainable solely by our results.

## Conclusions

Persistent moderate to heavy drinking and a higher cumulative alcohol consumption burden might increase the risk of AF, even in young adults. Young adults with heavy drinking habits should be educated about the hazardousness of AF and its association with heavy drinking.

## Notes

Supplement.
eMethods
**eTable 1.** Diagnostic ICD-10 Codes Used for Defining Covariates **eTable 2.** Subgroup Analyses **eTable 3.** Sensitivity Analysis: The Risk of AF in Subjects Without Prior MI, HF or Stroke at the Index Date and During the Follow-Up Period **eTable 4.** Sensitivity Analysis: The Risk of Atrial Fibrillation According to the Baseline Drinking Habit and 4-Year Cumulative Burden of Moderate-to-Heavy Drinking According to US Drinking Scale **eFigure 1.** Study Design **eFigure 2.** Cubic Spline Curve on the Association of the 4-Year Alcohol Consumption and the Incidence of Atrial Fibrillation
