# Supplementary material for: Habitual Alcohol Intake and Risk of Atrial Fibrillation in Young Adults in Korea
Source: JAMA Netw Open. 2022 Sep 2;5(9):e2229799. doi: 10.1001/jamanetworkopen.2022.29799 (PMC9440398; doi:10.1001/jamanetworkopen.2022.29799)
Supplement: Supplement. — eMethods eTable 1. Diagnostic ICD-10 Codes Used for Defining Covariates eTable 2. Subgroup Analyses eTable 3. Sensitivity Analysis: The Risk of AF in Subjects Without Prior MI, HF or Stroke at the Index Date and During the Follow-Up Period eTable 4. Sensitivity Analysis: The Risk of Atrial Fibrillation According to the Baseline Drinking Habit and 4-Year Cumulative Burden of Moderate-to-Heavy Drinking According to US Drinking Scale eFigure 1. Study Design eFigure 2. Cubic Spline Curve on the Association of the 4-Year Alcohol Consumption and the Incidence of Atrial Fibrillation [file jamanetwopen-e2229799-s001.pdf]

Supplemental Online Content

Han M, Lee S-R, Choi EK, et al. Habitual alcohol intake and risk of atrial fibrillation in young adults in Korea. *JAMA Netw Open*. 2022;5(9):e2229799. doi:10.1001/jamanetworkopen.2022.29799

eMethods
eTable 1. Diagnostic ICD-10 Codes Used for Defining Covariates
eTable 2. Subgroup Analyses
eTable 3. Sensitivity Analysis: The Risk of AF in Subjects Without Prior MI, HF or Stroke at the Index Date and During the Follow-Up Period
eTable 4. Sensitivity Analysis: The Risk of Atrial Fibrillation According to the Baseline Drinking Habit and 4-Year Cumulative Burden of Moderate-to-Heavy Drinking According to US Drinking Scale
eFigure 1. Study Design
eFigure 2. Cubic Spline Curve on the Association of the 4-Year Alcohol Consumption and the Incidence of Atrial Fibrillation

This supplemental material has been provided by the authors to give readers additional information about their work.

© 2022 Han M et al. *JAMA Network Open.*

eMethods

Calculation of the amount of alcohol consumption

The questionnaire used to investigate the alcohol intake of examinees in the Korean health examination is as follows. (Translated into English).

Please read the following questions and answer them according to your current situation.<br><br>On average, how many days per week do you drink alcohol?<br>☐0 ☐1 ☐2 ☐3 ☐4 ☐5 ☐6 ☐7<br><br>How much do you usually drink per day when you drink (in the number of glass(es))? _______<br><br>(※ Count by the glass used for each type of liquor. One can of beer (355 cc) is equivalent to 1.6 glasses of beer.)

The most consumed alcoholic beverages in Korea are beer and Soju (Korean traditional liquor). In general, beer glasses used in Korea are 220mL and Soju glasses are 50mL. Calculating the alcohol content of beer as 4.5% and that of Soju as 17%, and the specific gravity of alcohol as 0.8, one glass of beer in Korea contains 7.9g of alcohol (220*0.045*0.8=7.92) and one glass of Soju contains 6.8g of alcohol (50*0.17*0.8=6.8). By averaging the amount of alcohol in each liquor, we assumed that one standard drink (one glass of any type of liquor) contains approximately 7.5g of alcohol.

© 2022 Han M et al. *JAMA Network Open.*

eTable 1. Diagnostic ICD-10 Codes Used for Defining Covariates

Covariates	ICD-10-CM code and definition	
**Hypertension**	I10-I13, I15;<br>plus ≥1 prescription of anti-hypertensive drugs (thiazide, loop diuretics, aldosterone antagonist, alpha-/beta-blocker, calcium-channel blocker, angiotensin-converting enzyme inhibitor, angiotensin II receptor blocker).	ICD code≥1 time during hospitalization or<br>≥2 times in the outpatient clinic
**Diabetes mellitus**	E11-E14;<br>plus ≥1 prescription of anti-diabetic drugs (sulfonylureas, metformin, meglitinides, thiazolidinediones, dipeptidyl peptidase-4 inhibitors, α-glucosidase inhibitors, and insulin).	
**Dyslipidemia**	E78	
**Heart failure**	I50	
**Prior MI**	I21, I22	
**PAD**	I70, I73	
**COPD**	J41-44	
**Thyroid disease**	E03, E05	
**Sleep apnea**	G473	
**Prior ischemic stroke**	I60-62	
**CKD**	eGFR<60ml/min/1.73m^2	Based on the results of index (4th) health exam

* MI stands for myocardial infarction; PAD, peripheral artery disease; COPD, chronic obstructive pulmonary disease; CKD, chronic kidney disease; and eGFR, estimated glomerular filtration rate.

© 2022 Han M et al. *JAMA Network Open.*

eTable 2. Subgroup analyses

Subgroup		4-year alcohol burden	n	AF	IR per 1000	HR (95% CI)	P for interaction
Age	20-29	0	443,576	549	0.23	1 (Reference)	0.29
		1	106,440	159	0.28	1.07 (0.89-1.28)	
		2	74,367	109	0.27	0.98 (0.79-1.21)	
		3	67,863	114	0.31	1.06 (0.86-1.30)	
		4	66,343	120	0.33	1.08 (0.88-1.34)	
	30-39	0	445,806	963	0.38	1 (Reference)	
		1	96,934	286	0.52	1.18 (1.03-1.35)	
		2	73,720	204	0.48	1.06 (0.91-1.23)	
		3	76,160	247	0.56	1.20 (1.04-1.39)	
		4	86,627	315	0.63	1.32 (1.16-1.51)	
Sex	Male	0	529,317	1,107	0.38	1 (Reference)	0.07
		1	164,050	409	0.45	1.18 (1.06-1.33)	
		2	129,024	300	0.42	1.07 (0.94-1.22)	
		3	131,883	344	0.46	1.18 (1.03-1.32)	
		4	145,825	432	0.53	1.29 (1.15-1.44)	
	Female	0	360,065	405	0.20	1 (Reference)	
		1	39,324	36	0.17	0.88 (0.62-1.24)	
		2	19,063	13	0.13	0.68 (0.39-1.18)	
		3	12,140	17	0.26	1.40 (0.85-2.32)	
		4	7,145	3	0.08	0.44 (0.14-1.38)	
Hypertension	No	0	841,452	1,317	0.28	1 (Reference)	0.22
		1	187,110	376	0.36	1.15 (1.03-1.30)	
		2	133,546	248	0.33	1.01 (0.88-1.16)	
		3	126,700	294	0.41	1.20 (1.06-1.37)	
		4	131,093	337	0.46	1.29 (1.13-1.46)	
	Yes	0	47,930	195	0.73	1 (Reference)	
		1	16,264	69	0.76	1.03 (0.78-1.35)	
		2	14,541	65	0.80	1.06 (0.80-1.41)	
		3	17,323	67	0.69	0.91 (0.68-1.20)	
		4	21,877	98	0.80	1.04 (0.81-1.34)	

© 2022 Han M et al. *JAMA Network Open.*

eTable 2. Subgroup analyses (continued)

DM	No	0	873,476	1,471	0.30	1 (Reference)	0.56
		1	198,874	432	0.39	1.15 (1.03-1.28)	
		2	144,097	297	0.37	1.02 (0.90-1.16)	
		3	139,898	344	0.44	1.16 (1.02-1.31)	
		4	148,212	410	0.49	1.24 (1.10-1.39)	
	Yes	0	15,906	41	0.47	1 (Reference)	
		1	4,500	13	0.52	1.00 (0.53-1.88)	
		2	3,990	16	0.72	1.31 (0.72-2.35)	
		3	4,125	17	0.74	1.29 (0.72-2.30)	
		4	4,758	25	0.95	1.55 (0.92-2.60)	
DL	No	0	820,270	1,363	0.30	1 (Reference)	0.98
		1	184,162	394	0.39	1.15 (1.03-1.29)	
		2	132,364	274	0.37	1.05 (0.92-1.20)	
		3	127,373	309	0.43	1.16 (1.02-1.32)	
		4	133,772	369	0.49	1.26 (1.11-1.42)	
	Yes	0	69,112	149	0.39	1 (Reference)	
		1	19,212	51	0.48	1.08 (0.78-1.49)	
		2	15,723	39	0.45	0.96 (0.67-1.38)	
		3	16,650	52	0.56	1.16 (0.84-1.60)	
		4	19,198	66	0.62	1.221(0.90-1.65)	
COPD	No	0	862,946	1,451	0.30	1 (Reference)	0.78
		1	198,036	433	0.40	1.16 (1.04-1.29)	
		2	144,264	304	0.38	1.05 (0.92-1.19)	
		3	140,448	348	0.44	1.16 (1.03-1.31)	
		4	149,362	419	0.50	1.25 (1.12-1.41)	
	Yes	0	26,436	61	0.41	1 (Reference)	
		1	5,338	12	0.40	0.81 (0.43-1.51)	
		2	3,823	9	0.42	0.77 (0.38-1.58)	
		3	3,575	13	0.64	1.13 (0.61-2.11)	
		4	3,608	16	0.78	1.27 (0.71-2.28)	

© 2022 Han M et al. *JAMA Network Open.*

eTable 2. Subgroup analyses (continued)

Sleep apnea	No	0	888,461	1,509	0.31	1 (Reference)	0.99
		1	203,042	443	0.39	1.14 (1.03-1.27)	
		2	147,829	313	0.38	1.04 (0.92-1.18)	
		3	143,781	361	0.45	1.16 (1.03-1.31)	
		4	152,702	433	0.51	1.25 (1.12-1.40)	
	Yes	0	921	3	0.59	1 (Reference)	
		1	332	2	1.09	1.87 (0.30-11.59)	
		2	258	0	0	-	
		3	242	0	0	-	
		4	268	2	1.34	2.60 (0.39-17.35)	
Thyroid	No	0	871,038	1,446	0.30	1 (Reference)	0.68
		1	200,790	436	0.39	1.16 (1.04-1.29)	
		2	146,511	305	0.37	1.04 (0.92-1.18)	
		3	142,689	353	0.44	1.17 (1.04-1.32)	
		4	151,760	423	0.50	1.25 (1.12-1.40)	
	Yes	0	18,344	66	0.64	1 (Reference)	
		1	2,584	9	0.62	0.66 (0.32-1.36)	
		2	1,576	8	0.92	0.91 (0.43-1.95)	
		3	1,334	8	1.06	0.96 (0.45-2.07)	
		4	1,210	12	1.77	1.46 (0.76-2.82)	
MI	No	0	888,884	1,506	0.30	1 (Reference)	0.996
		1	203,225	443	0.39	1.14 (1.03-1.27)	
		2	147,954	312	0.38	1.04 (0.92-1.18)	
		3	143,928	361	0.45	1.17 (1.04-1.31)	
		4	152,899	435	0.51	1.26 (1.13-1.41)	
	Yes	0	498	6	2.18	1 (Reference)	
		1	149	2	2.42	1.08 (0.20-5.80)	
		2	133	1	1.34	0.79 (0.08-7.37)	
		3	95	0	0	-	
		4	71	0	0	-	

© 2022 Han M et al. *JAMA Network Open.*

eTable 2. Subgroup analyses (continued)

CHF	No	0	888,790	1,498	0.30	1 (Reference)	0.26
		1	203,215	444	0.40	1.15 (1.03-1.28)	
		2	147,997	310	0.38	1.03 (0.91-1.17)	
		3	143,922	360	0.45	1.17 (1.04-1.32)	
		4	152,865	434	0.51	1.26 (1.13-1.41)	
	Yes	0	592	14	4.39	1 (Reference)	
		1	159	1	1.20	0.22 (0.03-1.78)	
		2	90	3	6.09	1.02 (0.25-4.11)	
		3	101	1	1.84	0.35 (0.04-2.86)	
		4	105	1	1.77	0.35 (0.04-2.78)	
PAD	No	0	882,749	1,489	0.30	1 (Reference)	0.51
		1	201,918	438	0.39	1.14 (1.02-1.27)	
		2	147,088	308	0.38	1.03 (0.91-1.17)	
		3	143,043	358	0.45	1.17 (1.03-1.31)	
		4	151,945	433	0.51	1.26 (1.13-1.41)	
	Yes	0	6,633	23	0.63	1 (Reference)	
		1	1,456	7	0.87	1.37 (0.57-3.28)	
		2	999	5	0.90	1.47 (0.54-4.03)	
		3	980	3	0.55	0.77 (0.22-2.68)	
		4	1,025	2	0.35	0.57 (0.13-2.53)	
CKD	No	0	885,644	1,498	0.31	1 (Reference)	0.98
		1	202,750	442	0.39	1.15 (1.03-1.28)	
		2	147,697	312	0.38	1.04 (0.92-1.18)	
		3	143,708	359	0.45	1.16 (1.03-1.31)	
		4	152,633	433	0.51	1.26 (1.12-1.41)	
	Yes	0	3,738	14	0.66	1 (Reference)	
		1	624	3	0.85	0.87 (0.23-3.26)	
		2	390	1	0.45	0.57 (0.07-4.52)	
		3	315	2	1.14	1.16 (0.25-5.46)	
		4	337	2	1.03	0.96 (0.20-4.50)	

© 2022 Han M et al. *JAMA Network Open.*

eTable 2. Subgroup analyses (continued)

Prior stroke	No	0	888,842	1,505	0.31	1 (Reference)	0.996
		1	203,245	443	0.39	1.14 (1.03-1.27)	
		2	147,980	313	0.38	1.04 (0.92-1.18)	
		3	143,938	360	0.45	1.16 (1.03-1.31)	
		4	152,902	434	0.51	1.26 (1.12-1.41)	
	Yes	0	540	7	2.32	1 (Reference)	
		1	129	2	2.81	0.72 (0.14-3.76)	
		2	107	0	0	-	
		3	85	1	2.13	0.85 (0.10-7.24)	
		4	68	1	2.52	0.71 (0.08-6.35)	
BMI	<25	0	651,976	975	0.27	1 (Reference)	0.86
		1	131,040	245	0.34	1.09 (0.95-1.26)	
		2	89,653	155	0.31	0.94 (0.79-1.12)	
		3	82,581	177	0.38	1.09 (0.92-1.28)	
		4	83,178	199	0.42	1.15 (0.98-1.35)	
	≥25	0	237,406	537	0.41	1 (Reference)	
		1	72,334	200	0.50	1.19 (1.01-1.40)	
		2	58,434	158	0.49	1.13 (0.94-1.35)	
		3	61,442	184	0.54	1.21 (1.02-1.44)	
		4	69,792	236	0.61	1.33 (1.14-1.56)	
Smoking	Non	0	562,281	769	0.25	1 (Reference)	0.79
		1	80,389	132	0.30	1.14 (0.95-1.38)	
		2	42,648	69	0.29	1.07 (0.83-1.37)	
		3	30,600	56	0.33	1.13 (0.86-1.49)	
		4	21,268	47	0.40	1.28 (0.95-1.72)	
	Former	0	103,265	289	0.50	1 (Reference)	
		1	36,130	96	0.48	0.97 (0.77-1.22)	
		2	28,770	70	0.44	0.87 (0.67-1.13)	
		3	28,316	92	0.58	1.13 (0.89-1.42)	
		4	29,969	99	0.59	1.09 (0.86-1.37)	
	Current	0	223,836	454	0.36	1 (Reference)	

© 2022 Han M et al. *JAMA Network Open.*

		1	86,855	217	0.45	1.25 (1.06-1.46)	
		2	76,669	174	0.41	1.11 (0.93-1.32)	
		3	85,107	213	0.45	1.19 (1.01-1.40)	
		4	101,733	289	0.51	1.32 (1.14-1.53)	
Regular exercise	No	0	739,093	1,204	0.29	1 (Reference)	0.49
		1	165,299	354	0.39	1.15 (1.02-1.30)	
		2	119,659	249	0.37	1.05 (0.91-1.20)	
		3	116,807	291	0.44	1.18 (1.03-1.35)	
		4	124,918	357	0.51	1.29 (1.14-1.46)	
	Yes	0	150,289	308	0.37	1 (Reference)	
		1	38,075	91	0.44	1.07 (0.84-1.36)	
		2	28,428	64	0.41	0.95 (0.72-1.25)	
		3	27,216	70	0.46	1.03 (0.79-1.34)	
		4	28,052	78	0.50	1.06 (0.82-1.37)	

* n stands for number of participants; AF, atrial fibrillation; IR, incidence rate; HR, hazard ratio; DM, diabetes mellitus; DL, dyslipidemia; COPD, chronic obstructive pulmonary disease; MI, myocardial infarction; CHF, congestive heart failure; PAD, peripheral artery disease; CKD, chronic kidney disease; and BMI, body mass index.

© 2022 Han M et al. *JAMA Network Open.*

eTable 3. Sensitivity Analysis: The Risk of AF in Subjects Without Prior MI, HF or Stroke at the Index Date and During the Follow-Up Period

**4-year alcohol burden**	**n**	**AF**	**IR per 1000**	**HR (95% CI)**	**P-value**
0	887,798	1,485	0.30	1 (Reference)	0.0002
1	202,950	441	0.39	1.16 (1.04-1.29)	
2	147,763	309	0.38	1.04 (0.92-1.18)	
3	143,750	359	0.45	1.17 (1.04-1.32)	
4	152,733	433	0.51	1.27 (1.13-1.42)	

* n stands for number of participants; AF for atrial fibrillation; IR for incidence rate; HR for hazard ratio; and CI for confidence interval.

© 2022 Han M et al. *JAMA Network Open.*

eTable 4. Sensitivity Analysis: The Risk of Atrial Fibrillation According to the Baseline Drinking Habit and 4-Year Cumulative Burden of Moderate-to-Heavy Drinking According to US Drinking Scale

				**HR (95% CI)**	
	**No. of participants**	**AF (n)**	**IR (1000PY)**	**Crude**	**Adjusted model***
**Baseline drinking habit**					
Men					
None	257,661	554	0.39	1 (Reference)	1 (Reference)
Moderate	704,309	1,601	0.41	1.04 (0.95-1.15)	1.01 (0.92-1.12)
Heavy	135,904	403	0.53	1.37 (1.20-1.55)	1.26 (1.11-1.44)
				P=<.0001	P=0.0002
Women					
None	226,493	266	0.21	1 (Reference)	1 (Reference)
Moderate	176,651	176	0.18	0.88 (0.72-1.06)	0.92 (0.76-1.12)
Heavy	33,976	27	0.15	0.70 (0.47-1.04)	0.79 (0.53-1.19)
				P=0.1253	P=0.4374
**The 4-year alcohol burden (1 point to each moderate to heavy drinking during 4 consecutive years)**					
Men					
0	808,104	1,774	0.40	1 (Reference)	1 (Reference)
1	138,875	347	0.45	1.14 (1.01-1.27)	1.11 (0.99-1.24)
2	73,781	189	0.46	1.16 (1-1.35)	1.11 (0.96-1.29)
3	47,655	140	0.53	1.33 (1.12-1.58)	1.25 (1.05-1.48)
4	29,459	108	0.66	1.66 (1.37-2.02)	1.52 (1.25-1.85)
				P=<.0001	P=<.0001
Women					
0	359,424	400	0.20	1 (Reference)	1 (Reference)
1	39,318	36	0.17	0.84 (0.59-1.17)	0.89 (0.63-1.26)
2	19,076	13	0.13	0.62 (0.36-1.08)	0.69 (0.39-1.20)
3	12,151	17	0.26	1.27 (0.78-2.07)	1.43 (0.87-2.34)
4	7,151	3	0.08	0.39 (0.12-1.21)	0.45 (0.14-1.40)
				P=0.1113	P=0.1743

*The adjusted model was adjusted for age, sex, diabetes mellitus, hypertension, dyslipidemia, chronic obstructive pulmonary disease, sleep apnea, thyroid disease, myocardial infarction, heart failure, peripheral artery disease, chronic kidney disease, prior stroke, body mass index, smoking, performing regular exercise, and low income.

AF stands for atrial fibrillation; IR, incidence ratio; PY, person-year; HR, hazard ratio; and CI, confidence interval.

© 2022 Han M et al. *JAMA Network Open.*

eFigure 1. Study Design

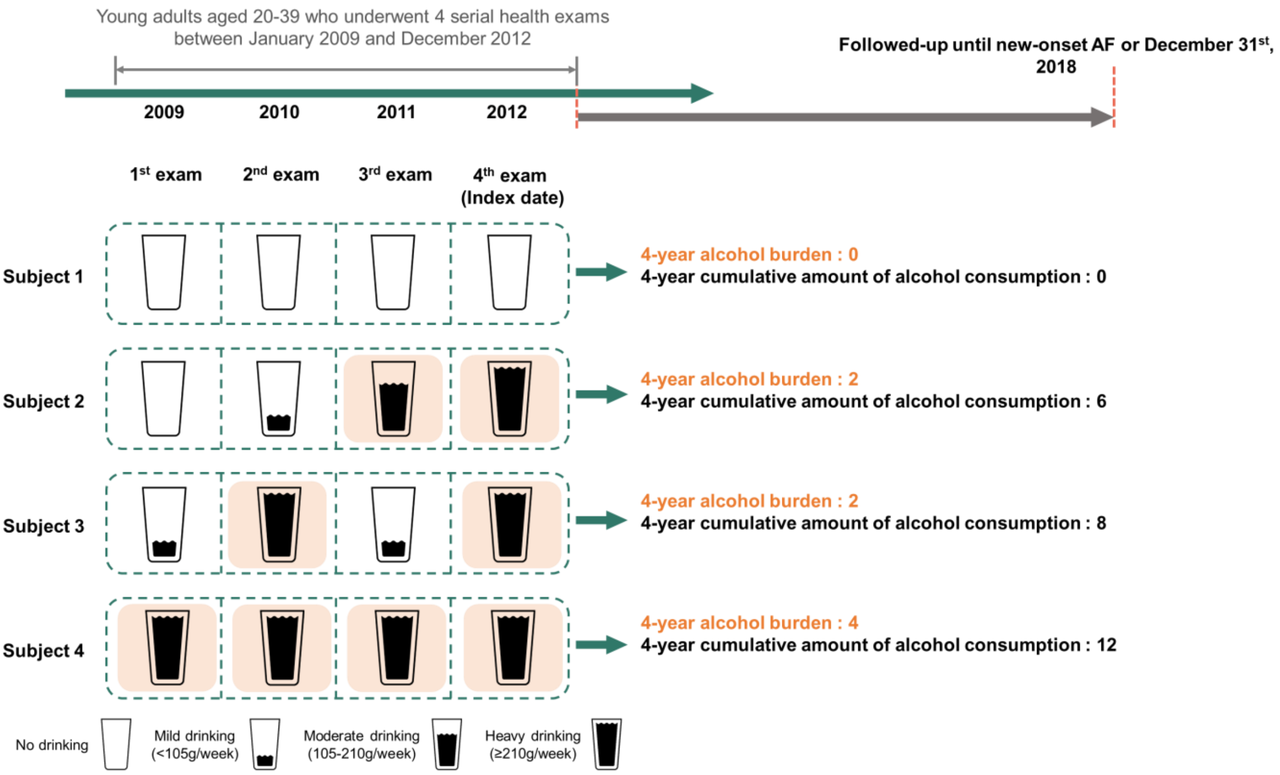

We defined less than 105 g, more than 210 g, and 105 g to 210 g of weekly alcohol consumption as mild, heavy, and moderate drinking, respectively. To operationally define the 4-year alcohol burden, one point was assigned to each moderate to heavy drinking (> 105 g/week, colored orange in the figure).

Additionally, to evaluate the dose-response relationship between the amount of alcohol consumption and risk of AF, a more stratified scoring was conducted. The novel semi-quantitative 4-year cumulative amount of alcohol consumption was calculated by assigning one, two, and three points for mild, moderate, and heavy drinking, respectively, using the previously established definitions (mild drinking, < 105 g/week, moderate, 105-210 g/week, and heavy, ≥ 210 g/week). The subjects were then categorized into 13 groups according to the semi-quantitative cumulative alcohol consumption burden for four years (0-12).

* Abbreviations: AF, atrial fibrillation

© 2022 Han M et al. *JAMA Network Open.*

eFigure 2. Cubic Spline Curve on the Association of the 4-Year Alcohol Consumption and the Incidence of Atrial Fibrillation

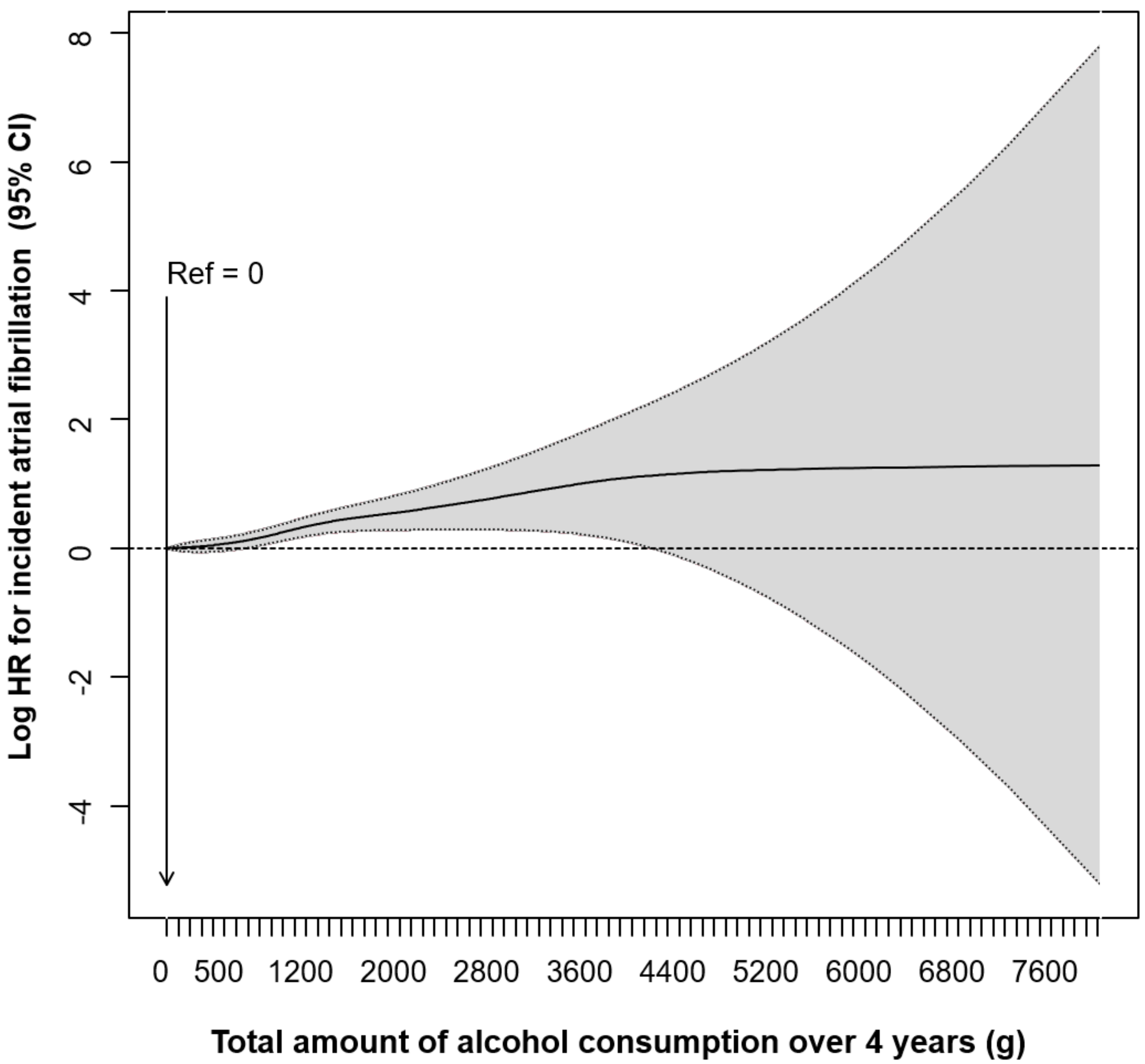

The alcohol consumption between 700 g and 4,200 g, which is approximately 90 to 560 standard drinks according to our categorization, and 50 to 300 standard drinks based on US standards, was associated with an increased risk of atrial fibrillation. The hazard ratio was not statistically significant when the alcohol consumption was greater than 4,200 g, presumably because the number of corresponding was too small.

* HR stands for hazard ratio; CI for the confidence interval.

© 2022 Han M et al. *JAMA Network Open.*
